# Retraction: Role of EAST-6 in renal injury by regulating microRNA-155 expression via TLR4/MyD88 signaling pathway in mice with Mycobacterium tuberculosis infection

**DOI:** 10.1042/BSR-20170021_RET

**Published:** 2020-09-28

**Keywords:** Early secretory antigenic target-6, MicroRNA-155, Mycobacterium tuberculosis, Myeloid differentiation factor 88, Renal injury, Toll-like receptor 4

This article is being retracted from Bioscience Reports at the request of the Editor-in-Chief and the Editorial Board following receipt of a notification from a reader, alerting the Editorial Board to duplicated Western blots in Figure 3C, which have appeared in multiple publications by different author groups.

The authors have been contacted in regards to the retraction, and have not responded to the Journal's queries and the concerns raised. The Editorial Board stand by the decision to retract given the concerns with the figures.

